# Supplementary material for: Myxinidin-Derived Peptide against Biofilms Caused by Cystic Fibrosis Emerging Pathogens
Source: Int J Mol Sci. 2023 Feb 4;24(4):3092. doi: 10.3390/ijms24043092 (PMC9964602; doi:10.3390/ijms24043092)
Supplement: Supplementary file 1 [file ijms-24-03092-s001.zip › ijms-2187960-supplementary.pdf]

Supporting information

Myxinidin-Derived Peptide against Biofilms Caused by Cystic Fibrosis Emerging Pathogens

Rosa Bellavita [1,†], Angela Maione [2,†], Simone Braccia [1], Marica Sinoca [2], Stefania Galdiero [1], Emilia Galdiero [2] and Annarita Falanga [3,*]

[1] Department of Pharmacy, School of Medicine, University of Naples 'Federico II', Via Domenico Montesano 49, 80131 Naples, Italy
[2] Department of Biology, University of Naples 'Federico II', Via Cinthia, 80126 Naples, Italy
[3] Department of Agricultural Sciences, University of Naples 'Federico II', Via dell' Università 100, 80055 Portici, Italy
* Correspondence: annarita.falanga@unina.it; Tel.: +39-081-253-4525
† These authors contributed equally to this work.

Table of contents

1. HPLC chromatograms and analytical data of peptides **WMR-1, WMR-2, WMR-3, WMR-4, WMR-5** (Figures S1–S5)

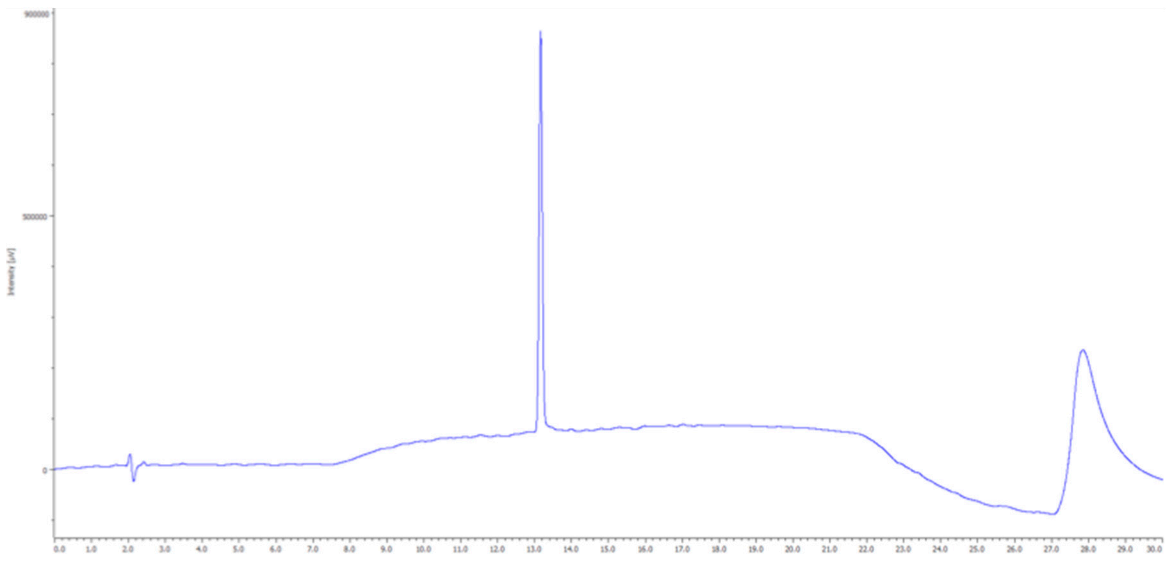

Figure S1. Chromatogram of peptide WMR-1 obtained by an analytical Jasco LC-NetII/ADC) equipped with a Phenomenex Kinetex C18 column (150 mm × 4.6 mm, 5 µm, 100 Å), and monitored by UV detection at 220 nm. [linear gradient 5–70% MeCN (0.1% TFA) in H_2O (0.1% TFA) over 20 min, flow rate of 1 mL/min]. Calculated mass: 1759.08. Found mass: $[M+2H]^+/2$ = 880.61.

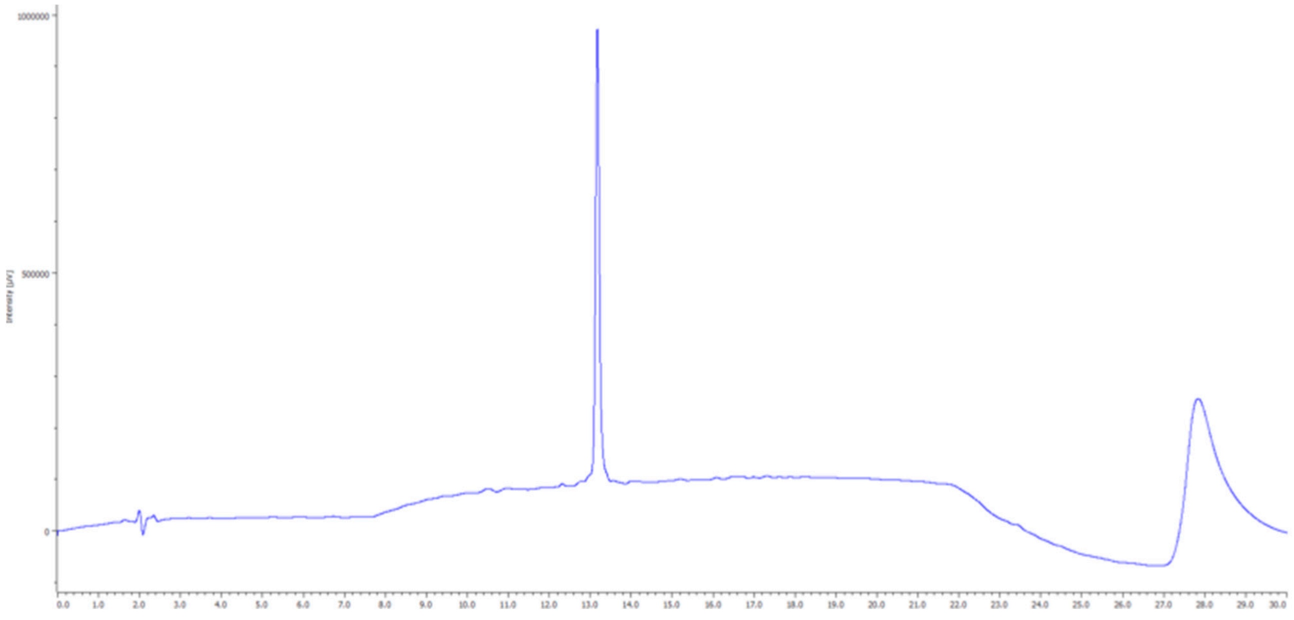

Figure S2. Chromatogram of peptide WMR-2 obtained by an analytical Jasco LC-NetII/ADC) equipped with a Phenomenex Kinetex C18 column (150 mm × 4.6 mm, 5 µm, 100 Å), and monitored by UV detection at 220 nm. [linear gradient 5–70% MeCN (0.1% TFA) in H_2O (0.1% TFA) over 20 min, flow rate of 1 mL/min]. Calculated mass: 1759.08. Found mass: $[M+2H]^+/2$ = 880.61.

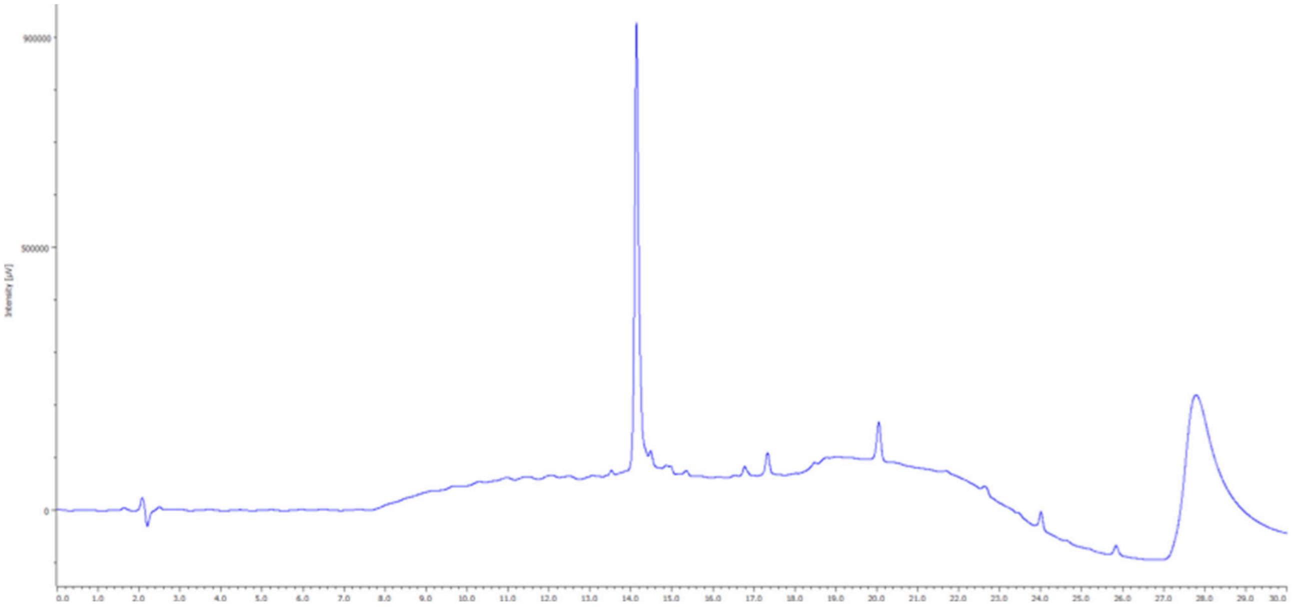

Figure S3. Chromatogram of peptide WMR-3 obtained by an analytical Jasco LC-NetII/ADC) equipped with a Phenomenex Kinetex C18 column (150 mm × 4.6 mm, 5 µm, 100 Å), and monitored by UV detection at 220 nm. [linear gradient 5–70% MeCN (0.1% TFA) in H_2O (0.1% TFA) over 20 min, flow rate of 1 mL/min]. Calculated mass: 1788.19. Found mass: $[M+2H]^{+}/2$ = 895.95.

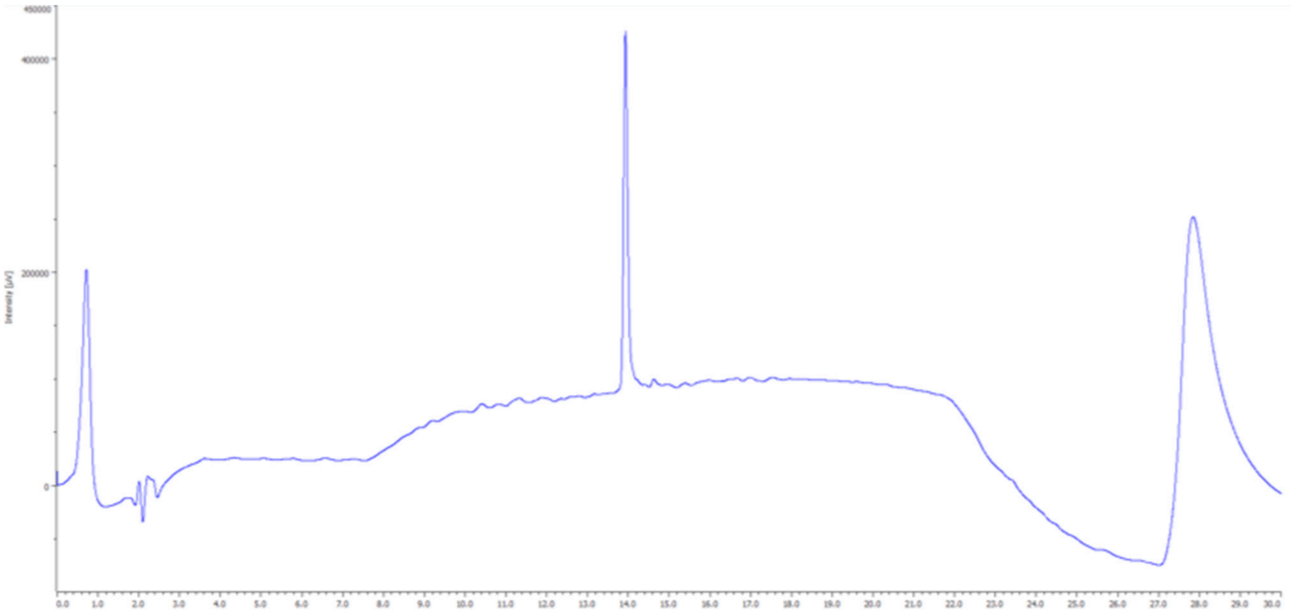

Figure S4. Chromatogram of peptide WMR-4 obtained by an analytical Jasco LC-NetII/ADC) equipped with a Phenomenex Kinetex C18 column (150 mm × 4.6 mm, 5 µm, 100 Å), and monitored by UV detection at 220 nm. [linear gradient 5–70% MeCN (0.1% TFA) in H_2O (0.1% TFA) over 20 min, flow rate of 1 mL/min]. Calculated mass: 1788.19. Found mass: $[M+2H]^{+}/2$ = 895.95.

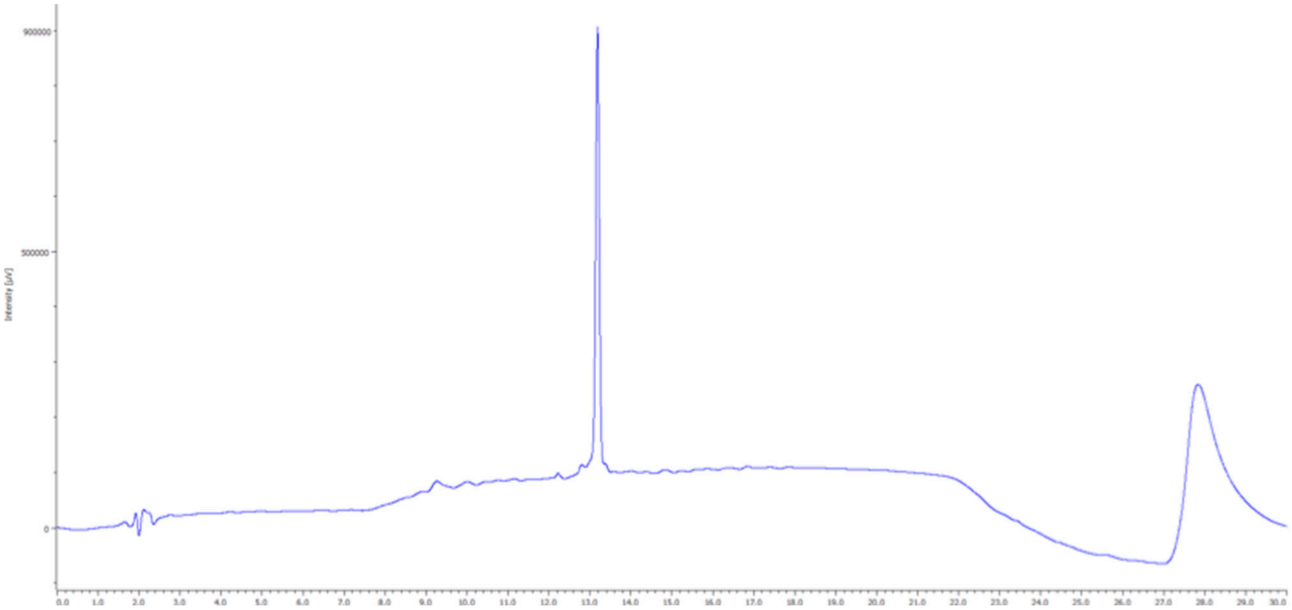

Figure S5. Chromatogram of peptide WMR-5 obtained by an analytical Jasco LC-NetII/ADC) equipped with a Phenomenex Kinetex C18 column (150 mm × 4.6 mm, 5 µm, 100 Å), and monitored by UV detection at 220 nm. [linear gradient 5–70% MeCN (0.1% TFA) in H_2O (0.1% TFA) over 20 min, flow rate of 1 mL/min]. Calculated mass: 1717.04 Found mass: $[M+2H]^{+}/2$ = 859.96.
